# Does single blind peer review hinder newcomers?

**DOI:** 10.1007/s11192-017-2264-7

**Published:** 2017-03-03

**Authors:** Marco Seeber, Alberto Bacchelli

**Affiliations:** 1 Ghent, Belgium; 2 Mekelweg 4, 2628 CD Delft, The Netherlands

**Keywords:** Double blind review, Single blind review, Bias, Newcomers, In-group out-group, Computer science

## Abstract

Several fields of research are characterized by the coexistence of two different peer review modes to select quality contributions for scientific venues, namely double blind (DBR) and single blind (SBR) peer review. In the first, the identities of both authors and reviewers are not known to each other, whereas in the latter the authors’ identities are visible since the start of the review process. The need to adopt either one of these modes has been object of scholarly debate, which has mostly focused on issues of fairness. Past work reported that SBR is potentially associated with biases related to the gender, nationality, and language of the authors, as well as the prestige and type of their institutions. Nevertheless, evidence is lacking on whether revealing the identities of the authors favors reputed authors and hinder newcomers, a bias with potentially important consequences in terms of knowledge production. Accordingly, we investigate whether and to what extent SBR, compared to a DBR, relates to a higher ration of reputed scholars, at the expense of newcomers. This relation is pivotal for science, as past research provided evidence that newcomers support renovation and advances in a research field by introducing new and heterodox ideas and approaches, whereas inbreeding have serious detrimental effects on innovation and creativity. Our study explores the mentioned issues in the field of computer science, by exploiting a database that encompasses 21,535 research papers authored by 47,201 individuals and published in 71 among the 80 most impactful computer science conferences in 2014 and 2015. We found evidence that—other characteristics of the conferences taken in consideration—SBR indeed relates to a lower ration of contributions from newcomers to the venue and particularly newcomers that are otherwise experienced of publishing in other computer science conferences, suggesting the possible existence of ingroup–outgroup behaviors that may harm knowledge advancement in the long run.

## Introduction

Peer review is the evaluation process employed by the largest majority of scientific outlets to select quality contributions (Bedeian 2004). It is a practice highly institutionalized and conferring legitimacy (DiMaggio and Powell 1983). However, in recent decades, researchers provided empirical evidence on the limitations of peer review, related, among others, to reviewers’ biases (Armstrong 1997), low inter-reviewers agreement (Bornmann and Daniel 2009), and weak capability to identify break through and impactful ideas (Campanario 2009; Chen and Konstan 2010; Siler et al. 2015). Thus, understanding how the organization of the peer review process can affect its outcomes is of crucial importance.

In recent years, scholars have begun exploring how different modes of organizing peer review can affect the quality of review and its outcome, such as testing the effects of incorporating monetary rewards for reviewers (Squazzoni et al. 2013) and variations in the number of reviewers (Roebber and Schultz 2011; Bianchi and Squazzoni 2015; Snell 2015).

Scholars have also investigated the consequences of adopting peer review modes with different visibility criteria concerning the authors’ identity, particularly double blind peer review (DBR)—where authors’ identities are disclosed only after acceptance of a paper—and single blind peer review (SBR)—where authors’ identities are visible throughout the entire review process. These studies, predominantly motivated by considerations of fairness, found that when authors’ identities are revealed to reviewers, evaluation is less objective and biases due to gender, nationality, and language, as well as the prestige and type of institution of affiliation play a role (Snodgrass 2006). However, supporters of SBR argue that the identity of authors is useful to judge the reliability of scientific claims, resulting beneficial to the advancement of knowledge (Pontille and Torny 2014). Thus, the debate between supporters of DBR and SBR review has been to some extent a dialogue of the deaf, the former stressing issues of fairness and the latter focusing on functionalistic arguments, which implicitly justify un-blinding for the superior interest of the advancement of knowledge.

However, by reviewing studies on innovation it can be argued that anonymity of authors can be beneficial for scientific advancement as well. In fact, studies of innovation highlight the importance of certain characteristics of the social context for both collective and individual propensity to innovate. In teams, newcomers are essential to raise new questions and provide new ideas, perspectives, and methods (Perretti and Negro 2007). Research on networks of innovation show that, at the individual level, the propensity of entrepreneurs towards innovation or reproduction of old ideas is influenced by the diversity of social relationships in which they are embedded (Marsden 1987; Ruef 2002). In a similar vein, studies of research activity have shown the detrimental effects of academic inbreeding (i.e., the tendency of academic institutions to recruit personnel that have studied in the same institution) on individual and institutional creativity and performance (Pelz and Andrews 1966; Soler 2001; Horta et al. 2010; Franzoni et al. 2014). Scientific outlets are a pivotal source of new inputs and ideas and, in the case of conferences, they are also social contexts where a community of scholars meet to develop relationships and future collaborations. It is thus of crucial importance to explore whether and to what extent a specific peer review mode introduces a bias against people that can bring new ideas and perspectives, namely early researchers or researchers that are new to that specific venue: *newcomers*.

In this article, we explore the hypothesis that when the identity of the authors is revealed, referees’ evaluation tend to be affected by authors’ past productivity. We investigate whether scientific outlets adopting a SBR display less contributions from researchers that have less publications in general and in the same outlet, than DBR outlets. We also explore whether SBR outlets display a larger share of contributions from researchers that are relatively new to the outlet but otherwise productive. In particular, we test two competing hypotheses, that in SBR contribution from these researchers are: (1) more frequent because overall productivity positively affects reviewers’ evaluation, either that are (2) less frequent because reviewers might be skeptical of contributions from researchers that comes from other venues, or even perceive them as a potential competitors threating their academic tribe (Becher and Trowler 1989/2001). We test these hypotheses on a sample of 21,535 research papers published 71 among the 80 most impactful conferences in the field of computer science research. This empirical context is particularly suitable, as SBR and DBR are both widely adopted by computer science conferences.

The main contribution of the article is thus twofold: (1) we stress the implication for knowledge production of a newcomers’ bias in peer review, and (2) explore bias towards two types of newcomers and their interaction. Empirically, we consider a large sample of articles and venues, thus providing a stronger evidence on the impact on individual reputation bias in SBR (Snodgrass 2006).

The article is organized as follows: In the following section, we review the scholarly debate on anonymity and related bias in peer review, selected studies on innovation and inbreeding, as well as formulate the hypotheses. Subsequently, we introduce the data and method of analysis, and in the fourth section we present the analysis and the results. We conclude discussing the findings and directions of future research.

## Theoretical framework

### Peer review process anonymity and biases

Pontille and Torny recently described how peer review practice and the debate on anonymity in peer review have evolved throughout time (Pontille and Torny 2014). At its outset, in the 18th century, peer review was organized in the form of an editorial committee that collegially examined and selected manuscripts, while the editor took the main responsibility for the final decision (Crane 1967; Bazerman 1988). Only in the last century, due to the increasing specialization of science and growth of research production, the use of external reviewers diffused as to complement the competences of the editorial boards (Burnham 1990). From the ’50 a debate emerged regarding the anonymity of authors to reviewers. Sociologists first spotted that article’s assessment should regards its content and not be affected by the reputation and prestige of its authors or their institutions of affiliation. Quest for anonymity were backed by the Mertonian norms of Science^1^ and, in particular, by the norm of universalism, stating that scientific claims should be evaluated according to the same impersonal criteria’, regardless of personal or social attributes of the author (Merton 1973). From mid-1970s, anonymization of authors spread to journals in management, economy, and psychology as well, due to studies examining reviewers’ “bias” (Zuckerman and Merton 1971; Mahoney 1977), as well as pressures from women within American learned societies, which highlighted the low acceptance rates of articles by female scholars (Benedek 1976; Weller 2001).^2^

Opposed to the view that the evaluation of scientific writings must be based only on the content of the article, other scholars argued that to validate a scientific claim the reviewers needs to link writings to writers, because the credit that reviewers give to experiments and results is also backed by past studies and the use of specific equipment, so that anonymization would weaken evaluation (Ward and Goudsmit 1967). Opponents to anonymization where also skeptical on the effectiveness of anonymity as such, since authors’ self-quotations would disclose their identities or reviewers would try still to attribute a text to an author (BMJ 1974). These arguments were particularly popular in the experimental sciences, so that anonymization of authors did not diffuse in fields like physics, medicine, biology and biomedicine as much as in the social sciences (Weller 2001). Moreover, in recent years access to search engines have arguably made easier to guess authors’ identity, so that some journals in the field of Economics decided to return to SBR (AER 2011).

Parallel to the debate on anonymity of authors is the discussion on the opportunity to disclose reviewers’ identities. In turn, four main categories of peer review can be identified based on (non-)anonymity of reviewers and authors: both unknown (double blind), authors known and reviewers unknown (single blind), authors unknown and reviewers known (blind review) and both known (open peer review). A survey of 553 journals from eighteen disciplines found that DBR is the most diffused peer review mode (58%) and of growing diffusion, followed by SBR (37%) and open review (5%) (Bachand and Sawallis 2003).

So far, empirical studies related to anonymity of reviewers have focused on three main issues, namely the efficacy of blinding, quality of reviews, and potential biases. As to the efficacy of blinding, research across a wide range of disciplines found that blinding is effective in most of the cases (53–79%) (Snodgrass 2006).

Evidence on the quality of reviews in the two modes is mixed (Snodgrass 2006).

Studies on bias in peer review have focused on four main topics, namely: (i) error in assessing true quality, (ii) social characteristics of the reviewer, (iii) content of the submission and (iv) social characteristics of the author (Lee et al. 2013).

Since SBR and DBR differ for revealing or not the author’s identity, then research comparing SBR and DBR has mostly focused on the latter typology of bias, namely when an author’s submission is not judged solely on the merit of the work, but related to her/his academic rank, sex, place of work, publication record, *etc.* (Peters and Ceci 1982). Budden *et al.* provided evidence of a gender bias by showing that a journal that switched to DBR experiences an increased representation of female researchers (Budden et al. 2008). However, their findings were contested (Webb et al. 2008) and most research on the subject did not find evidence of a gender bias when authors’ identity is revealed (Lee et al. 2013; Blank 1991; Borsuk et al. 2009). Instead, there is consistent evidence on bias related to the prestige of the institution to which authors are employed (Peters and Ceci 1982), language, namely in favor of authors from English speaking countries (Ross et al. 2006), and nationality, with journals favoring authors from the same country of the journal (Daniel 1993; Ernst and Kienbacher 1991), whereas there is mixed evidence on whether American reviewers tend to favor or be more critical with compatriots (Link 1998; Marsh et al. 2008). An affiliation bias has been detected when reviewers and authors/applicants enjoy formal and informal relationships (Wenneras and Wold 1997; Sandström and Hällsten 2008), although not always leading to more positive evaluations (Oswald 2008). Only two studies analyzed the influence of individual productivity, and they do not reach a consensus (Snodgrass 2006). In particular, a study of two conferences found no impact on prolific authors (Madden and DeWitt 2006), while a further analysis on the same data using medians rather than means, reached the opposite conclusion (Tung 2006).

### The importance of newcomers

The capability of given social structures to hinder or ease access to newcomers has important implications for innovation, research, and knowledge advancement. The importance of newcomers for innovation has been highlighted by several studies. Katz argued that newcomers represent a novelty-enhancing condition in teams, as they challenge and broaden the scope of existing methods and knowledge, whereas when members of a group remain stable, over time they tend to reduce external communication, to ignore and isolate from critical sources of feedback and information (Katz 1982). Since agents search for solutions within a limited range of all possible alternatives, then homogeneous groups will search within a similar range (Perretti and Negro 2007). On the contrary, newcomers contribute to innovation by bringing new knowledge and also by searching opportunities and feedbacks in new directions (McKelvey 1997), so that higher incidence of newcomers is predictive of team innovativeness (Perretti and Negro 2007).

Literature on organizational learning (Levitt and March 1988; March 1991) shows that the mixing newcomers and established members affects organizational learning and innovation. According to March, experienced members know more on average, but their knowledge is redundant with that already in the organization (March 1991). New recruits, instead, are less knowledgeable than the individuals they replace, but what they know is less redundant and they are more likely to deviate from it. Newcomers enhance exploration, innovation, and the chances of finding creative solutions to team problems, whereas old-timers increase exploitation, inertial behavior, and resistance to new solutions.

Overall, renewing members maintains social communities innovative, by easing access to information, improving ability to consider alternatives, and generating novel and creative solutions (Bantel and Jackson 1989; Jackson 1996; Watson et al. 1993; Guzzo and Dickson 1996). In the case of research activity, novelty and creativity are crucial. Research on ‘academic inbreeding’, e.g. the tendency of academic institutions to recruit personnel that have studied in the same institution has shown its several drawbacks, for individuals as well as research institutions, related to the parochialism of an inbred faculty, which are much less likely than non-inbred colleagues to exchange scholarly information outside their group (Berelson 1960; Pelz and Andrews 1966; Horta et al. 2010).

Similarly to academic institutions, scientific outlets are social spaces committed to the production of knowledge. They represent both crucial sources of new ideas and, in the case of conferences, are also social contexts where a community of scholars meet and establish new collaborations. It is thus important to understand whether different peer review modes ease or hinder access of newcomers.

### Hypotheses

We explore the conjecture that when the identity of the authors is revealed to referees, their evaluation will be affected by the authors’ previous productivity—in that specific venue and/or overall hindering publications from newcomers.

Accordingly, our first expectation is that, compared to DBR outlets, the SBR outlets will display relatively less contributions from researchers that have less experience in publishing in that outlet and overall.

*Hp1 outlet newcomers* A scientific outlet’ share of articles from researchers with few or no publications in the outlet is smaller when contributions are selected via SBR rather than DBR other outlets’ characteristics being the same.

*Hp2 overall newcomers* A scientific outlet’ share of articles from researchers with few or no publications overall is smaller when contributions are selected via SBR rather than DBR other outlets’ characteristics being the same.

Since revealing the identity is expected to hinder publications from newcomers to the venue and newcomers overall, then the effect of un-blinding is uncertain regarding a particular category of newcomers, namely experienced newcomers’: authors that are newcomers to the outlet but which have published elsewhere. Two different expectations can be formulated.

First, while experienced newcomers can be disadvantaged for they might not be sufficiently acquitted to theories, methods and approaches in the outlet’ area of study, referees in SBR might take into account their origin and, when newcomers are particularly experienced, then their reputation may support the validity of their claims. According to this line of reasoning we can formulate the following hypothesis.

*Hp 3a Experienced newcomers welcomed* A scientific outlet’ share of articles from researchers new to the outlet but with experience of publishing in other outlets, will be larger when contributions are selected via SBR rather than DBR other outlets’ characteristics being the same.

A competing hypothesis is that reviewers might be prejudiced towards contributions coming from other areas of research and/or they might perceive experienced researchers coming from other venues as a potential threat to their academic tribe’ (Becher and Trowler 2001); accordingly:

*Hp 3b Experienced newcomers not welcomed* A scientific outlet’ share of articles from researchers new to the venue but with experience of publishing in other venues will be smaller when contributions are selected via SBR rather than DBR other outlets’ characteristics being the same.

## Data and methods

### Sample

The field of computer science research is particularly suitable to address the questions and hypotheses of this article, since DBR and SBR are both widely adopted. Computer science research is mostly oriented to propose new models, algorithms, or software, so that reviewers typically focus on a paper’s novelty, whether it addresses a useful problem and the solutions is applicable in practice as well as based on sufficient theoretical and empirical validation (Ragone et al. 2013). Differently from most research fields, in computer science research the conferences are considered at least as important as journals as a publication venue (Meyer et al. 2009; Chen and Konstan 2010; Freyne et al. 2010). Peer review for computer science conferences is done on submitted full papers, as opposed to other academic fields where the selection of contributions is often done on (extended) abstracts. This is due to the importance of conferences for computer science academic research.

The peer review is often done by a committee of known reviewers (aka, program committee). The assignment of the submitted papers to reviewers is facilitated by a bidding process: The reviewers bid on articles they would prefer to review; reviewers are expected to review articles for which they feel competent and for which they have no conflict of interest; this process is applied both with DBR and SBR. Based on the bidding information, the submissions are assigned to reviewers, who will remain anonymous to the authors. Online or physical program committee meetings take place to discuss the inclusion of each submitted contribution into the conference program and proceedings.

As subjects of our study, we consider the 21,535 research papers (and their 47,201 authors) published in 2014 or 2015 in the proceedings of 71 of the 80^3^ largest computer science conferences in terms of the cumulative number of citations received (source: Microsoft academic search^4^). We retrieved information on conferences’ size as well as reputation from Microsoft academic search (a free public search engine for academic papers and literature, developed by Microsoft^5^) and we extracted information on peer review mode from the conferences’ websites. As our subjects, we only considered research papers, thus excluding conference contributions such as tool demonstrations, tutorials, short papers, posters, keynote speeches.

To collect historical information on authors in computer science, we used DBLP,^6^ the computer science bibliography. DBPL is the largest database on academic publications on computer science research, it indexes more than 32,000 journal volumes, 31,000 conference or workshop proceedings, and 23,000 monographs, for a total of 3.3 million publications published by more than 1.7 million authors. The full dataset was retrieved on 23rd March 2016 from the publicly available full DBLP data dump.^7^

For each author of the aforementioned 21,535 research papers, we used these data to build a profile based on past productivity in the venues and in the field of computer science overall (Table 2 provides additional details).

### Tests and variables

We aim to explore whether a conference’ share of contributions from different types of newcomers is predicted by articles being reviewed under a SBR or a DBR mode (article level characteristic) and selected conference characteristics.

To define whether an article was written by newcomers, we considered the productivity of the most prolific co-author before 2014 (or 2015). Accordingly, we first computed percentiles of past productivity at conference level and in the field of computer science (considering publications in conferences and journals included in DBLP) Table 1. Next, we defined as conference newcomers the authors with a past productivity in the conference up to the 25th percentile, i.e., as maximum two publications. In a similar vein, we considered as field newcomers those authors with a past productivity in the field of computer science research up to the 25th percentile, i.e., maximum 41 publications. Further, we defined as experienced newcomers the authors that are newcomers to the conference (below 2 publications) and experienced of publishing in other venues, namely having a productivity in the field above the median of the sample (above 85 publications).

**Table 1:** Number of articles before 2014 of the most productive co-author, in the 71 most impactful conferences (*Source*: DBLP and Microsoft Academic Search)

	Percentile 05	Percentile 25	Median	Mean	Percentile 75
Venue	0	2	7	12	16
Total	9	41	85	131	169

By employing values at article level, we could compute the dependent variables at conference level, namely proportions given by the ration between the number of articles from newcomers and the total number of articles accepted (Table 2). The dependent variables are then given by the average of $n_j$ binary variables $y_i$, assuming a value 1 if the article is authored by newcomer(s) and 0 if not, where $n_j$ is the total number of articles accepted in the conference *j*, so that the proportion $\pi _j$ results from $n_j$ independent events of peer review and $y_j$ are binary variables that can be modelled through a logistic regression.

1 $$\begin{aligned} \pi _j = \sum _{1}^{n_j}\frac{y_j}{n_j} \end{aligned}$$

Table 2 describes the dependent variables.

**Table 2:** Dependent variables—*Source*: authors’ elaboration on DBLP data

Variable name	Description
Share conference newcomers	Number of articles authored by conference newcomers divided by the total number of articles accepted to the conference
Share field newcomers	Number of articles authored by field newcomers divided by the total number of articles accepted to the conference
Share experienced newcomers	Number of articles authored by newcomers to the conference but experienced of research in the field divided by the total number of articles accepted to the conference

The independent variables include: (i) the peer review mode, the (ii) age and the (iii) reputation of the conference (Table 3). In the hypotheses section we have already discussed the expected effects of peer review mode. Moreover, older conferences are expected to display a smaller share of contributions from newcomers, as the community around the conference is expected to stabilize over time. The reputation and quality of the conference may also have a negative impact on the share of newcomers, since newcomers can be discouraged submitting to a high reputed conference and high quality conference tend to be more selective, thus hindering less experienced researchers. We consider the conference size as control variable.^8^

Table 3 describes the characteristics of the predicting variables.

**Table 3:** Independent and control variables—*Source*: authors’ elaboration on Microsoft Academic search data and conference website information

Variable name	Description
Peer review mode	Whether articles in a conference are reviewed in DBR or DBR
Age conference-gm	The number of editions of the conference—centred on the grand mean of the sample
Reputation conference-gm	As a proxy we employ the Field Rating indicator from Microsoft academic search. This indicator is similar to the h-index (Hirsch. 2005). Therefore. a conference with a Field Rating *h* has published *h* papers each of which has been cited in other papers at least *h* times. The indicator only considers publications and citations within a field. thus showing the impact of the conference within that specific field The values are centred on the grand mean of the sample
Size conference-gm	The number of articles accepted to the conference—centred on the grand mean of the sample

We run logistic regressions of proportions, where $Logit(\pi _j)$ represents the predicted proportion of articles from newcomers to conference *j*, $\beta _0$ represents the log odds of being an article authored by newcomers for a conference adopting single blind peer review, and of grand mean of age, reputation and size (the reference categories), while parameters $\beta _1 \cdot DBR_j$, $\beta _2 \cdot Age_j$, $\beta _3 \cdot Rep_j$, and $\beta _4 \cdot Size_j$ represent the differentials in the log odds of being a paper from newcomers for a paper reviewed in double blind peer review, presented in a conference of $age_{j-grand mean}$, $reputation_{j-grand mean}$, and $size_{j-grand mean}$.

2 $$\begin{aligned} Logit(\pi _j) = \beta _0 + \beta _1 \cdot DBR_{j-gm} + \beta _2 \cdot Age_{j-gm} + \beta _3 \cdot Rep_{j-gm} + \beta _4 \cdot Size_{j-gm} \end{aligned}$$

We estimate the model through Bayesian Markov Chain Monte Carlo methods (Snijders and Bosker 2012), which produce chains of model estimates and sample the distribution of the model parameters. As a diagnostic for model comparison we employ the Deviance Information Criterion (DIC), which penalizes for a model complexity—similarly to the Akaike Information Criterion (AIC)^9^ and it is a measure particularly valuable for testing improved goodness of fit in logit models (Jones and Subramanian 2012).

## Results

### Descriptive statistics

Descriptive statistics can be provided both at conference level and article level. Table 4 shows that *on average*, the share of contributions from newcomers to a conference is 32%, from field newcomers is 23%, whereas contributions from experienced newcomers represent 10%. There is a considerable level of variations between conferences, as shown by standard variations, minimum and maximum values. In our set, 34 conferences adopt DBR and 36 SBR; SBR conferences tend to be larger, so that 66% of the published articles were reviewed in SBR. Considerable variability exists regarding conferences’ age, their reputation, and size.

**Table 4:** Conferences characteristics—descriptive statistics - n. 71

Variable name	Minimum	Maximum	Mean	SD
Share conference newcomers	0.06	0.72	0.32	0.14
Share field newcomers	0.01	0.65	0.23	0.12
Share experienced newcomers	0	0.43	0.10	0.08
Double blind	0	1	0.48	0.50
Age conference	5	56	26.9	10.9
Reputation conference	43	182	94	29
Size conference	22	2018	303	370

Some significant correlations emerge between conferences’ characteristics (Table 5). Most notably, high reputed conferences have less contributions from newcomers and larger conferences have less contributions from experienced newcomers. Considering conference averages, there is no significant correlation between the peer review mode and shares of newcomers. However, simple correlations do not take into account of other conferences characteristics. Moreover, a macro-macro association (between share of newcomers and peer review mode) is inappropriate to draw meaningful implication for a micro-micro relationship—i.e., that an article from newcomers has more chances to be accepted under DBR—because it would incur in an ecological fallacy, i.e., the relationship between individual variables cannot be inferred from the correlation of the variables collected for the group to which those individuals belong (Robinson 2009).

**Table 5:** Pearson’s correlations between conferences’ characteristics—n. 71

	Share conf. newcomers	Share field newcomers	Share experienced newcomers	Double blind	Age conf.	Reputation conf.	Size
Share conf. newcomers	1.000	0.204	0.690**	−0.013	−0.088	−0.227	−0.196
Share field newcomers	0.204	1.000	−0.390**	−0.151	0.016	−0.245*	0.177
Share experienced newcomers	0.690**	−0.390**	1.000	0.093	−0.105	−0.055	−0.234*
Double blind	−0.013	−0.151	0.093	1.000	0.220	0.345**	−0.237*
Age conf.	−0.088	0.016	−0.105	0.220	1.000	0.391**	−0.357**
Reputation conf.	−0.227	−0.245*	−0.055	0.345**	0.391**	1.000	−0.092
Size	−0.196	0.177	−0.234*	−0.237*	−0.357**	−0.092	1.000

Article level correlations (Table 6) indeed show a significant and positive association between DBR and the article being coauthored from newcomers, although only for conference newcomers and experience newcomers the correlation is positive, as expected, whereas it is negative for field newcomers.

**Table 6:** Correlations article characteristics—no. 21.535

	Conference newcomers	Field newcomers	Experienced newcomers	Double blind
Conference newcomers	1.000	0.360**	0.450**	0.071**
Field newcomers	0.360**	1.000	−0.167**	−0.036**
Experienced newcomers	0.450**	−0.167**	1	0.090**

### Hypotheses 1 and 2

Logistic regressions of proportions are the appropriate technique to explore whether conferences adopting DBR are more likely to display larger proportion of contributions from newcomers, while taking into consideration other conferences’ characteristics.

Tables 7 and 8 present the results of the regressions exploring hypotheses 1 and 2. For each hypothesis, the results of three regression models are displayed: (i) an empty model—e.g., a model with no predicting variables, (ii) DBR model—e.g., a model with only the peer review mode as predicting variable, (iii) full model, including all the predicting variables.

**Table 7:** Regression share of newcomers to the conference

	Conference newcomers								
	Empty model			DBR model			Full model		
		S.E.	Sig.		S.E.	Sig.		S.E.	Sig.
Cons	−0.91	0.02	***	−1.02	0.02	***	−0.92	0.03	***
Double blind				0.33	0.03	***	0.40	0.04	***
Age conference-gm							−0.0031	0.0018	
Reputation conference-gm							−0.0084	0.0006	***
Size-gm							−0.00042	0.00003	***
DIC	2071.02			1964.17			1550.77

**Table 8:** Regression share of newcomers to computer science

	Computer science newcomers								
	Empty model			DBR model			Full model		
		S.E.	Sig.		S.E.	Sig.		S.E.	Sig.
Cons	−1.08	0.02	***	−1.02	0.02	***	−1.06	0.03	***
Double blind				−0.17	0.03	***	0.01	0.04	
Age conference-gm							0.0024	0.0018	
Reputation conference-gm							−0.0080	0.0006	***
Size-gm							−0.00011	0.00003	***
DIC	1882.39			1857.65			1679.55		

The results confirm hypothesis 1: DBR is a significant predictor of a higher share of articles from newcomers to the conference. To calculate the odds of being an article from newcomers to the conference for DBR compared to the baseline SBR, we exponentiate the differential logit, thus: $\exp(0.40)=1.50$, which means 50% more chances in case of DBR than SBR. The age of the conference has not a significant impact, whereas more reputed and larger conferences display relatively less contributions from newcomers to the conference. *DIC* values highlight the better fit of the full model in respect to DBR model and empty model.

Hypothesis 2 is not confirmed: DBR is not a significant predictor of a higher share of articles from newcomers to the overall field of computer science research, e.g., authors with relatively less publications on DBLP. The age of the conference has not a significant impact, whereas more reputed and larger conferences display relatively less contributions from newcomers to the field.

### Hypotheses 3a and 3b

The results of the regression predicting the share of contributions from experienced newcomers support the hypothesis 3b ‘experienced newcomers not welcomed’ (Table 9). DBR is in fact a significant and positive predictor of a higher share of articles from this category of newcomers. The odds of being an article from experienced newcomers for DBR compared to the baseline SBR is: $\exp (0.49)=1.64$, which means 64% more chances in case of DBR than SBR. The age and size of the conference have a significant and negative impact, whereas reputation is not significant. *DIC* values highlight the better fit of the full model in respect to the DBR model and the empty model.

**Table 9:** Regression share of experienced newcomers

	Experienced newcomers								
	Empty model			DBR model			Full model		
		S.E.	Sig.		S.E.	Sig.		S.E.	Sig.
Cons	−2.51	0.03	***	−2.79	0.04	***	−2.54	0.05	***
Double blind				0.69	0.05	***	0.49	0.07	***
Age conference-gm							−0.0126	0.0032	***
Reputation conference-gm							−0.0011	0.0010	
Size-gm							−0.00066	0.00007	***
DIC	1451.44			1278.53			1161.55		

To further explore the relationship between peer review mode and contributions from experienced newcomers we provide descriptive statistics of the share of contribution from four categories of authors: (i) newcomers in computer science and in the conference, (ii) newcomers in computer science and experienced in the conference, (iii) experienced in computer science and in the conference, (iv) experienced in computer science and newcomers in the conference. The threshold for the conference is at 25th percentile of productivity (below 3 publications), whereas we considered three different thresholds of productivity for defining an author as experienced in computer science research, namely: (1) above 25th percentile (41 publications), (2) above median (85 publications) and (3) above 75th percentile (169 publications).

Table 10 confirms that, compared to SBR conferences, the DBR conferences display a larger share of contributions from newcomers to the conference (categories i and iv), in particular those that are experienced in computer science research and especially highly experienced ones, as the share of contributions for experienced newcomers is 19% in DBR versus 12% in SBR for experienced above 25th percentile, 11 versus 6% above median and 5 versus 2% above 75th percentile of productivity. In turn, DBR conferences display almost two times more contributions from highly experienced newcomers than SBR conferences.

**Table 10:** Number and share of contributions from categories of coauthors

	Computer science experienced >25th percentile	Conference newcomer <25 percentile	SBR	DBR	SBR	DBR
			Count	Count	%	%
(i)	Newcomer	Newcomer	2.066	1.059	14	15
(ii)	Newcomer	Experienced	1.733	598	12	8
(iii)	Experienced	Experienced	8.767	4.217	61	58
(iv)	Experienced	Newcomer	1.739	1.356	12	19

	Computer science experienced >median	Conference newcomer <25 percentile	SBR	DBR	SBR	DBR
			Count	Count	%	%
(i)	Newcomer	Newcomer	2.970	1.624	21	22
(ii)	Newcomer	Experienced	4.452	1.729	31	24
(iii)	Experienced	Experienced	6.048	3.086	42	43
(iv)	Experienced	Newcomer	835	791	6	11

	Computer science experienced >75th percentile	Conference newcomer <25 percentile	SBR	DBR	SBR	DBR
			Count	Count	%	%
(i)	Newcomer	Newcomer	3.463	2.056	24	28
(ii)	Newcomer	Experienced	7.477	3.160	52	44
(iii)	Experienced	Experienced	3.023	1.655	21	23
(iv)	Experienced	Newcomer	342	359	2	5
			14.305	7.230	100	100

### Alternative specifications of newcomers

As a final test, we explore whether the results are confirmed with more stringent definitions of newcomers. We test different thresholds, namely:

- newcomers to the conference as researchers with (i) no previous publications, (ii) maximum one publication;
- newcomers to the field as researchers with maximum 18 publications (e.g. 10th percentile of productivity);
- newcomers to the conference and experienced in the field considering newcomers those researchers with maximum one publication to the conference and experienced in the field those researchers with productivity above the median value.

The alternative specifications are highly correlated with the previous ones. Conferences’ share of newcomers to the conference (below 3 publications) correlates at 0.812** with newcomers conference 0 publications and 0.964** with newcomers conference maximum one publication; shares of newcomers to the field below 25th and 10th percentile of productivity correlate at 0.930**; the two measures of conferences’ share of newcomers to the conference and experienced in the field correlate at 0.956**.

The results of the regressions confirm the findings (Table 11).

**Table 11:** Full model binary logistic regressions with alternative specifications

	Conference newcomer: no publications			Conference newcomer: max 1 publications		
		S.E.	Sig.		S.E.	Sig.
Cons	−2.05	0.04	***	−1.34	0.03	***
Double blind	0.30	0.06	***	0.30	0.05	***
Age conference-gm	−0.001	0.003		−0.001	0.0020	
Reputation conference-gm	−0.008	0.0009	***	−0.008	0.0007	***
Size-gm	−0.00040	0.00005	***	−0.00042	0.00004	***
DIC	1451.44			1161.55		
pD	4.97			5.02		

	Field newcomer <10th percentile (18 publications)			Experienced in the field (above median) and conference newcomer (<2 publications)		
		S.E.	Sig.		S.E.	Sig.
Cons	−2.10	0.04	***	−2.92	0.06	***
Double blind	−0.04	0.06		0.45	0.08	***
Age conference-gm	0.01	0.003	***	−0.008	0.004	*
Reputation conference-gm	−0.01	0.0009	***	0.0002	0.001	
Size-gm	−0.00012	0.00005	**	−0.00070	0.00008	***
DIC	1316.0			958.0		
pD	5.00			5.01		

*** $$p\,<\,0.001$$; ** $$p\,<\,0.01$$; * $$p\,<\,0.05$$

## Conclusion

This article investigated whether revealing the identity of authors to referees is related to shares of publications from newcomers, as referees’ evaluation may be affected by authors’ track record of publications. Understanding the effects of peer review modes on the accessibility to newcomers is important as newcomers are shown by literature on innovation and inbreeding in research to be important in providing new perspectives, novel and creative ideas and solutions, thus playing a crucial role for advancing knowledge in a given field of study.

We explored the assumption of a reputation bias in computer science research, where two modes of peer review are adopted, namely single blind and double blind peer review, where identity of authors is revealed to referees in the first but not in the latter mode. We considered 71 among the 80 most impactful computer science conferences, and retrieved data on 21,535 articles and conference characteristics from the DBLP database and conferences websites. We tested the hypotheses that three categories of newcomers are related to less publications in SBR in respect to DBR, namely newcomers to the conference, newcomers to computer science research, and newcomers to the conference that are otherwise experienced in publishing in computer science. We found that, after taking into consideration the size, age and reputation of the conference, the contributions from newcomers to the conference are underrepresented when articles are reviewed in SBR mode. We did not find a confirmation that contributions from newcomers to computer science research are hindered in SBR conferences, which can possibly be related to the fact that compared to DBR conferences, in SBR conferences the experienced researchers are underrepresented when they are newcomers to the conference. In fact, regression results and descriptive analysis show that DBR display almost two times more contributions from this category of authors. Overall, the results suggest that by knowing the identity of the authors, reviewers may be biased towards authors that are not sufficiently embedded in their research community. In recent years some journals decided to switch the peer review mode from DBR to SBR, under the argument that search engines have made easier to guess authors’ identity (AER 2011); our results suggest that at least identity is not made fully evident in all fields, so that reintroducing SBR may have non-negligible consequences in terms of access to newcomers and bias in peer review. Arguably, in order to consider whether our findings can be generalized to academic journals and other field of research, it has to be considered how easy is to guess or retrieve the authors’ identity, namely it can be expected that: (i) reviewers of academic journals focused on niche research topics are more likely to know who the authors are than reviewers of academic journals focused on broad research topics; and that (ii) in fields where it is common practice to publish pre-prints—for instance Economics, on websites like repec.org then reviewers can retrieve authors’ identity more easily than in fields where this is not a common practice.

We identify some promising directions for future research. First, to provide further evidence on the consequences of revealing authors’ identity on the outcome of peer review, future studies can consider longitudinal data and conferences that have switched peer review mode in the considered period. This will allow to test our or similar hypotheses with a multilevel design and to explore random effects as well (Subramanian et al. 2009). Availability of data on both submitted and accepted papers would allow additional evidence on this regards. Second, future studies may explore the extent and the way in which different degree of a conference openness to newcomers affect knowledge evolution and advancement in a research community.

## Footnotes

1 The norms are communalism, universalism, disinterestedness and organized skepticism—the so called CUDOS’ (Merton 1973).

2 Pontille and Torny built a detailed depiction on the evolution of anonymity debate (Pontille and Torny 2014).

3 9 were excluded for: (i) adopting a different review process than the bidding process typically employed in computer science (e.g. VLDB), (ii) some missing or not clear information (HICSS, ISCAS, ISMB), (iii) not found on DBLP (BIOMED, Storage and retrieval) or multiple pages (ECCV), (iv) one case of merge (IGCA in GECCO).

4 Top conferences in computer science by cumulative number of citations Microsoft academic search: http://academic.research.microsoft.com/RankList?entitytype=3&topdomainid=2&subdomainid=0&last=0&orderby=1.

5
http://academic.research.microsoft.com.

6
http://dblp.uni-trier.de/.

7 Dump of the data is available at: http://dblp.uni-trier.de/xml/; information and statistics on DBLP can be retrieved at http://dblp.uni-trier.de/faq/What+is+dblp.html and http://dblp.uni-trier.de/statistics/.

8 We also controlled whether conferences indexed in Scopus or the Web of Science (WoS) display different peer review modes or can predict the share of newcomers. However, we found that the large majority of conferences are indexed and no significant difference in the peer review mode along indexed and non-indexed conference. In fact only seven conferences are not indexed in Scopus, of which four are DBR and three SBR; 64 are indexed, of which 30 DBR and 34 SBR. Four conferences are not indexed in the WoS (one in DBR and three SBR), 11 have been covered but not updated to nowadays (9 in DBR and 2 in SBR), 56 are indexed (24 DBR and 32 SBR).

9 The Akaike Information Criterion—AIC (Akaike, 1974) compares models by considering both goodness of fit and complexity of the model, estimating loss of information due to using a given model to represent the true model, i.e. a hypothetical model that would perfectly describe the data. Accordingly, the model with the smaller AIC points out the model that implies the smaller loss of information, thus having more chances to be the best model. In particular, given n models from 1 to n models and $model_min$ being the one with the smaller AIC, then the exponential of: ($AIC_{min} AIC_j$)/2 indicates the probability of model *j* in respect to $model_min$ to minimize the loss of information.
